# Immunohistochemical detection of PD-L1 among diverse human neoplasms in a reference laboratory: observations based upon 62,896 cases

**DOI:** 10.1038/s41379-019-0210-3

**Published:** 2019-02-13

**Authors:** Dennis P. O’Malley, Yuhang Yang, Saskia Boisot, Sucha Sudarsanam, Jian-Feng Wang, Vladislav Chizhevsky, Guohua Zhao, Shehla Arain, Lawrence M. Weiss

**Affiliations:** Department of Pathology, NeoGenomics Laboratories, Aliso Viejo, CA USA

**Keywords:** Cancer microenvironment, Immune evasion

## Abstract

Targeting of the PD1/PD-L1 immune checkpoint pathway has rapidly gained acceptance as a therapeutic strategy for a growing number of malignancies. Testing for expression of PD-L1 in tumor cells and immune cells has been used as a companion or complementary test for drugs targeting the PD1/PD-L1 pathway. We evaluated the results of PD-L1 testing in a large reference lab cohort. Using Food and Drug Administration-approved methods and interpretive instructions for each individual test, 62,896 cases were evaluated for PD-L1 using antibody clone 22C3, 28-8, SP142, or SP263. Case data analyzed included test results and information on tumor location and clinical history. No clinical outcome information was available and no attempt was made to correlate PD-L1 results with any other tests performed. The following numbers of cases were evaluated: 22C3 with tumor proportion score [*n* = 52585], 22C3 with combined positive score [*n* = 2631], 28-8 [*n* = 4191], SP142 [*n* = 850], and SP263 [*n* = 70]. In 22C3/tumor proportion score cases, the general results were as follows: negative 33.1% (*n* = 17,405), (low) expression 33.9% (*n* = 17,822), and high expression 29.5% (*n* = 15,486). In cases identified as metastatic, the results were as follows: negative 35.9% (*n* = 1411), (low) expression 30.8% (*n* = 1211), and high expression 30.7% (*n* = 1208). We found broad ranges of expression in tumor types with increasing positivity, as adenocarcinomas were reported as poorly differentiated, whereas squamous cell carcinomas showed more positivity as tumors were described as well-differentiated. The results of many individual tumor types were evaluated and showed, in general, high levels of positive expression. Practical challenges and observations of PD-L1 stain results and interpretation are also discussed.

## Introduction

Programmed death 1 (PD1) is a cell surface receptor expressed on cytotoxic T cells and pro-B cells, which binds to its cognate ligands PD-L1 and PD-L2, expressed on macrophages, epithelial cells, and other normal cells. Under normal physiologic conditions, the PD1/PD-L1 interaction produces specific conformational changes, which protect normal cells from immune recognition, and inhibits subsequent destruction by cytotoxic T cells, which would otherwise lead to a state of autoimmunity. As a result of this inhibition, reactive T cells become exhausted through signaling pathways, which lead to a combination of cessation of division and proliferation, and programmed cell death or apoptosis [1–3].

Certain neoplasms have developed mechanisms of evading this immune surveillance by upregulating PD-L1 expression on the surface of neoplastic cells, such that their PD-L1 receptors may bind to the PD1 ligand on activated T cell, and ultimately render them inactive and subject to clonal exhaustion. In this way, the neoplastic cells are able to escape this so-called “immune checkpoint” and continue to proliferate unabated. In recent years, cancer immunotherapy has focused on the development of a new generation of immunotherapy agents, which specifically block the interplay between tumor cells and the immune system, known as immune checkpoint inhibitors, of which the PD1/PD-L1 axis is but just one target [4, 5]; other targets of interest include cytotoxic T lymphocyte-associated protein 4, lymphocyte-activation gene 3, and killer-cell immunoglobulin-like receptor [4–7].

There are currently five approved therapeutic agents on the market targeting the PD1/PD-L1 pathway, two of which (nivolumab and pembrolizumab) are humanized IgG monoclonal antibodies directed at the PD-1 receptor, whereas the other three (atezolizumab, durvalumab, and avelumab) are humanized IgG monoclonal antibodies directed at the PD-L1 receptor [Table 1]. Each of these drugs binds to a different epitope on their respective target and therefore each has a distinct immunogenic profile and, by extension, its own dynamic range. Based on results of large-scale clinical trials showing statistically significant response rates and improvements in overall survival in the context of a variety of solid tumors, including non-small cell lung carcinomas, gastrointestinal carcinomas, head and neck squamous cell carcinomas, renal cell carcinomas, urothelial carcinomas, cervical carcinomas, and breast carcinomas, as well as in lymphomas and melanoma, all have been approved for use as second-line treatment in patients whose tumors have stopped responding to conventional chemotherapy, whereas pembrolizumab has recently received approval for use as the first-line therapy for advanced/metastatic non-small cell lung carcinomas in which the tumor cells show > 50% PD-L1 expression, as it was shown to be associated with a significantly longer progression-free and overall survival with fewer adverse events than in patients receiving platinum-based chemotherapy [8–12].

**Table 1:** Summary of antibodies, targets, drugs, and medications associated with PD1/PD-L1 immune checkpoint therapy

Antibody	Drug	Drug target	Indication	Comments
22C3 (Dako)	Pembrolizumab (Keytruda®); Merck	PD1	NSCLC, melanoma, HNSCC, urothelial, gastric/GEJ, CHL, MSI-H/dMMR	
28-8 (Dako)	Nivolumab (Opdivo™); Bristol-Myers-Squibb	PD1	Advanced nsNSCLC, HNSCC, colorectal cancer (MSI-H/dMMR), melanoma, advanced liver cancer, CHL, advanced renal cancer, advanced urothelial cancer	Positive results = > 1%; nsNSCLC clinical results cutoffs at = > 1%; = > 5%; = > 10%
SP142 (Ventana)	Atezolizumab (Tecentriq™); Roche	PD-L1	Urothelial, NSCLC	
SP263 (Ventana)	Durvalumab (Imfinzi®); AstraZeneca	PD-L1	Urothelial, advanced NSCLC in patients whose disease has not progressed following platinum-based CRT	
73-10 (Dako)	Avelumab (Bavencio®); EMD Seronon/Pfizer	PD-L1	Merkel cell carcinoma, urothelial	Other tumor types in clinical trials

*CHL* classic Hodgkin lymphoma; *CRT* chemo-radiation therapy; *GEJ* gastroesophageal junction; *HNSCC* head and neck squamous cell carcinoma; *MSI-H/dMMR* microsatellite instability–high/deleted mismatch repair; *ns* non-squamous; *NSCLC* non-small cell lung cancer

The advent of personalized healthcare, which refers to developing targeted therapeutics for specific patients or patient subgroups by identifying which patients are most likely to experience a favorable benefit-risk outcome with a selected therapy, has necessitated the development of an array of in vitro laboratory tests designed to measure predictive biomarker levels in these patients, with a view to tailoring individual treatment protocols. These diagnostic assays fall into one of two distinct categories, companion diagnostics and complementary diagnostics, based on requirement for drug eligibility [13, 14]. Companion diagnostic tests provide information that is essential for use of each of the aforementioned immune checkpoint inhibitors, are typically linked to a specific drug within their approved label, and determine patient eligibility for treatment with the corresponding drug. Complementary diagnostic tests may assist in the therapeutic decision-making algorithm associated with a particular therapy by informing on which patients may benefit from that therapy, but they do not restrict patients from receiving co-developed therapies based on the outcome of the diagnostic test, because therapeutic benefit with that drug has been demonstrated in all patients, regardless of biomarker expression status. The first companion diagnostic test to receive Food and Drug Administration (FDA) approval was the Her2 in-situ hybridization assay for trastuzumab in 1998 and, although the term “complementary diagnostic” had been in used since the 1990s. The PD-L1 immunohistochemical assay for use with nivolumab was the first complementary diagnostic test to meet FDA regulatory requirements [13]. Both categories of tests can inform on enhanced benefits in subgroups of patients, depending on degree of biomarker expression at varying cutoffs, and matching PD-L1 biomarker assays have been developed for each of the aforementioned five immune checkpoint inhibitors, with each developed by different companies, run on different analytic platforms, and each requiring their own respective validation studies with some distinctive methods of scoring [15, 16] [Table 2].

**Table 2:** PD-L1 assay interpretation guidelines and scoring

Antibody	Site/tumor type	Interpretation guidelines [positive]^a^
22C3	Gastroesophageal	Combined positive score (CPS); [Tumor + immune cells = > 1%]
	Other sites	Tumor proportion score (TPS); [Tumor = > 1% expression; tumor = > 50% high expression]
28-8		[Tumor cells > 1%]
	NSCLC	PD-L1 expression [tumor = > 1%; tumor = > 5%; tumor = > 10%^b^]
	HNSCC	PD-L1 expression [Tumor cells > 1%]
	Urothelial	PD-L1 expression [Tumor cells > 1%]
	Melanoma	PD-L1 expression [Tumor cells > 1%]
SP142	Urothelial	PD-L1 expression [ = > 5% in tumor-infiltrating immune cells]
	NSCLC	PD-L1 expression [Tumor cell = > 50% or Immune cell = > 10%]
SP263	Urothelial	PD-L1 status [Tumor cells = > 25% OR Immune cells present > 1% and tumor associated immune cell staining = > 25% OR Immune cells present = 1% and tumor associated immune cell staining 100%: PD-L1 status – high]
	NSCLC	PD-L1 IHC score [Tumor cells = > 1%]

^a^Additional indications and interpretation methods have been approved since the time of data collection. These interpretations were not included in the evaluation of this data

^b^Different expression cutoffs are associated with different levels of survival with nivolumab therapy

This study is intended to be largely observational. In this study, we evaluate the ordering and expression patterns of various PD-L1 antibodies using their individual FDA-approved methodologies. In addition, we address individual tumor- and sample-type expression results. Finally, we examine some common pitfalls and challenges in PD-L1 immunohistochemical staining interpretation.

## Materials and methods

Materials were sent for consultation to Neogenomics Laboratories from multiple locations. Testing was performed at our laboratory in Aliso Viejo, California. In each individual case, testing was performed either as requested for specific PD-L1 testing, or as part of a comprehensive evaluation for diagnosis or prognostic/theranostic markers in a tumor. As is typical in reference laboratory testing, submitted clinical history was minimal in most cases and was limited to tumor site (in most cases) with some indication of general tumor type (often), or specific diagnosis either by text (occasional) or international calssification of diseases code (occasional). No clinical follow-up is available on individual results. Further, because of limitations of the scope of this research, no attempt was made to correlate PD-L1 results with any other tests performed. All research was performed in accord with local and national standards for ethical research.

Staining was performed using methods for each antibody, as designated in each FDA-approved reagent kit [Table 3], using four anti-PD-L1 antibodies as follows: 22C3 (Dako; Carpinteria, California), 28-8 (Dako; Carpinteria, California), SP142 (Ventana/Roche; Tucson, Arizona), and SP263 (Ventana/Roche; Tucson, Arizona). Each scoring pathologist had specific training for the specific antibody and particular indication for testing as appropriate [Table 2].

**Table 3:** Anti-PD1/PD-L1 antibody staining information

Antibody	Mfg.	Pretreatment	Mfg./Device	Reagent
22C3	Dako	EnVision FLEX	Dako Autostainer Link 48	Dako Linker
28-8	Dako	EnVision FLEX	Dako Autostainer Link 48	Dako Linker
SP142	Ventana/Roche	CC1 Cell Conditioning	Ventana BenchMark ULTRA	OptiView detection kit
SP263	Ventana/Roche	CC1 Cell Conditioning	Ventana BenchMark ULTRA	OptiView detection kit

Data searches were performed using a natural language search of submitted information for site and submitted clinical history. However, as an example a submitted site of “lung” and history of “cough” would not allow for thorough categorization. All cases may not be represented from larger data set due to ambiguous or missing information in submitted information. In many cases, the possibility of a primary or metastatic tumor (such as the lung, brain, or liver sites) could not be disambiguated. When possible, searches were performed with parameters that would include or exclude data in such a way as to make the results relatively unambiguous. However, rare cases that had unusual presentation (e.g., lung carcinoma metastatic to thyroid) may have been included in some search sets. In the case of the lung, two different search parameters were used, in an attempt to assess whether internal results were relatively consistent, in addition to obtain fairly “pure” results for lung cases.

Cases that were considered quantity not sufficient lacked appreciable tumor on the PD-L1 stain. Although FDA guidelines specify that stains should not be scored when there are < 100 tumor cells present, in practical terms, if appreciable aggregates or clusters of tumor were present on PD-L1 stain, scoring was attempted. Other causes for rejection included diffuse necrosis, diffuse granular staining without specific membrane staining, or unreadable tissue due to histologic limitations (wrinkles, folds, tissue fall-off, etc.).

Statistical evaluations were performed using https://www.socscistatistics.com/tests/chisquare2/ (November 2018) for simple *χ*^2^-analysis. Kruskal–Wallis and Dunn’s test were analyzed in R version 3.5 (R Foundation for Statistical Computing, Vienna, Austria. http://www.R-project.org/)

## Results

**Fig. 1:**
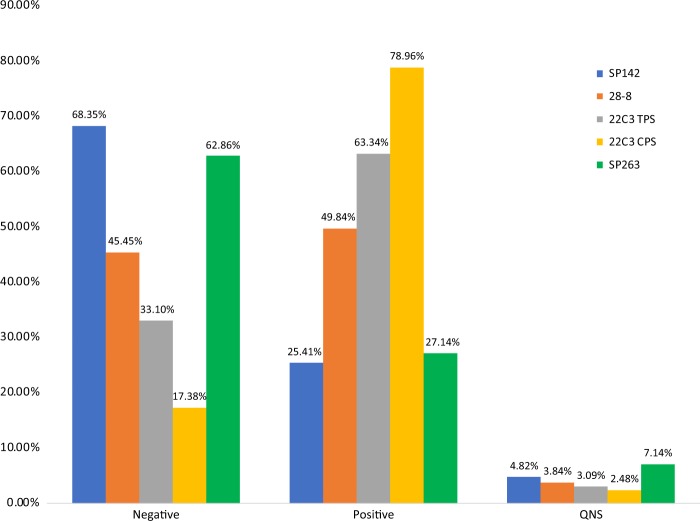
Combined results for positive expression, negative, and quantity not sufficient for SP142, 28-8, and 22C3 tumor proportion score (TPS), 22C3 combined positive score (CPS), and SP263

We evaluated results for a total of 62,896 cases (Fig. 1). Cases from February 2017 till May of 2018 were evaluated. Cases without any identification of gender, age, or as part of clinical trials were not further evaluated (*n* = 2577). When considering all evaluated cases, 3.7% (*n* = 2226) of cases were considered quantity not sufficient for analysis and no score result was generated. No specific additional analysis was performed on these cases. The male-to-female ratio of all tested cases was 52:48. The average age was 68.6 years (range < 1–105 years) [Table 4] [Supplementary Materials 1].

**Table 4:** Combined results of anti-PD-L1 testing

Antibody	Number of cases	Average age, years (range)	M:F	QNS number (%)
22C3				
TPS	52585	68.8 ( < 1–105)	51:49	1872 (3.6%)
CPS	2623	65.5 (51–78)	67:33	97 (3.7%)
28-8	4191	68.0 (2–103)	53:47	197 (4.7%)
SP142	850	69.0 (2–96)	48:52	53 (6.2%)
SP263	70	64.0 (2–94)	36:64	7 (10.0%)

*CPS* combined positive score; *QNS* quantity not sufficient; *TPS* tumor proportion score

As part of routine laboratory quality assurance practices, monthly scores for PD-L1 22C3 tumor proportion score (no expression, expressed, highly expressed), and combined positive score results (no expression, expression) were compared. In 7 months during that were analyzed, tumor proportion scores showed minimal month-to-month variation (percent positive range 61.9–66.2%) and combined positive scores showed slightly more variation (percent positive: 77.9–86.1%). [Supplemental materials 2]

### 22C3: combined positive score

Immunohistochemistry for PD-L1 using 22C3 with the combined positive score is intended for evaluation of gastric and gastroesophageal adenocarcinoma during the time of this study. In addition to this indication, a variety of cases were submitted for combined positive score scoring, irrespective of testing/therapeutic guidelines.

A total of 2623 cases were evaluated using 22C3/combined positive score. The results of 22C3/combined positive score are summarized in Table 5. The age range was 51–78 years (average 65.5) with a male-to-female ratio of 67:33. Quantity not sufficient cases accounted for 3.7% (*n* = 97). Esophageal and gastric cancers were comparable in the number of cases with expression (85.8% vs. 83.6%).

**Table 5:** Analysis of 22C3 combined pathology score (CPS) groups

Tumor type*	Search parameter	Number of cases	Average age, years	M:F	QNS %	Negative ( < 1%)	Expressed ( > 1%)
All cases		2623	65.5	1760:863	3.7	17.4	79.0
Esophageal	“esophageal” or “esophagus” or “GEJ” or “gastroesophageal”	1059	67.2	862:197	2.5	11.7	85.8
Gastric	“gastric” or “stomach” or “pyloric” or “antrum” or “cardia”	696	64.8	421:275	3.0	13.4	83.6
Adenocarcinoma	“adenocarcinoma”	729	64.5	514:215	3.6	16.5	80.0
Squamous cell carcinoma	“squamous” or “SCC” or “SCCA”	67	68.9	39:28	4.5	11.9	83.6
Metastatic	“metastatic”	158	62.9	92:66	6.3	25.3	68.3

### 22C3: tumor proportion score

**Fig. 2:**
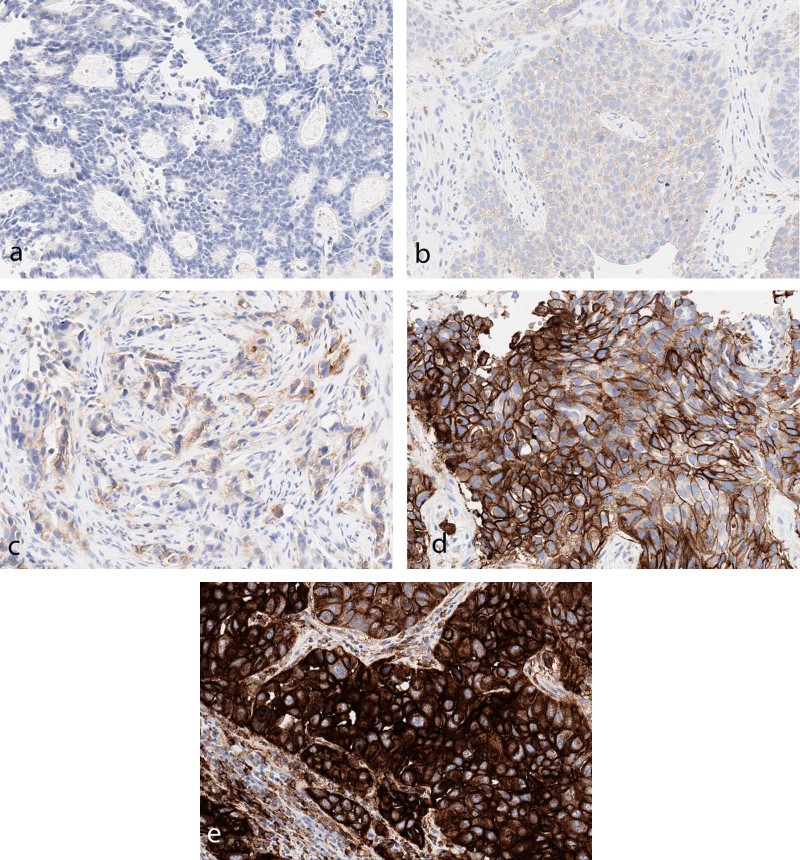
Scoring intensity of the majority of cells in the fields represented (22C3). **a** 0: negative. **b** 1 + : dim positive. **c** 2 + : moderate positive. **d** 3 + : strong positive. **e** 3 + : very strong positive; this latter pattern appears as uniform, intense membranous, and cytoplasmic staining. Although not specific, this may indicate constitutive overexpression of PD-L1 by tumor cells

A total of 52,585 cases were evaluated. The age range was < 1–105 years (average 68.8), with a male-to-female ratio of 51:49. Quantity not sufficient cases accounted for 3.6% (*n* = 1872); the quantity not sufficient percentages in all subset analysis was somewhat variable ( < 1–11.1%); however, those groups with the highest quantity not sufficient rates (Hodgkin lymphoma, thymoma) had only small numbers of cases. In 22C3/tumor proportion score scored cases, the general results were as follows: negative 33.1% (*n* = 17,405), (low) expression 33.9% (*n* = 17,822), and high expression 29.5% (*n* = 15,486) [Table 6]. Tumors that had highest numbers of no expression ( > 45% of cases) were as follows: neuroendocrine, endometrial, mucinous adenocarcinoma, well-differentiated adenocarcinoma, thyroid, bladder, and renal. Tumors with the largest numbers of highly expressed cases ( > 40% of cases) were as follows: pericardial fluid, mycosis fungoides, poorly differentiated adenocarcinoma, and adenosquamous carcinoma. Staining intensity was recorded for 22C3/tumor proportion score, but not analyzed further (Fig. 2).

**Table 6:** Analysis of 22C3 tumor proportion score (TPS) groups

Tumor type^a^	Search parameter	Number of cases	Average age, years	M:F	QNS %	Negative ( < 1%)	Expressed (1-49%)	Highly expressed ( = > 50%)
All cases		52,585	68.9	51:49	3.6	33.1	33.9	29.5
Brain	“astrocytoma” or “glioblastoma” or “gbm”	82	56.4	49:33	1.2	42.7	37.8	18.3
Breast	“breast” or “lobular” or [“ductal” not “pancreatic”]	737	61.3	0:737	3.7	58.2	27.5	10.6
Colon	“colon” or “colonic” or “sigmoid” or “rectum”	1142	64.3	321:521	1.9	66.6	26.2	5.3
Endometrial	“endometrium” or “endometrial” or “uterus”	258	63.5	0:258	3.1	54.7	38.0	4.3
Esophageal	“esophageal” or “esophagus” or “GEJ” or “gastroesophageal”	384	66.8	288:96	2.9	47.9	36.7	12.5
Gastric	“gastric” or “stomach” or “pyloric” or “antrum” or “cardia”	545	64.5	305:240	3.9	45.9	30.3	20.0
Gastrointestinal stromal tumor	[“gastrointestinal” and “stroma”] or “GIST”	42	65.0	24:18	2.4	50.0	45.2	2.4
Hodgkin lymphoma	“Hodgkin” and “lymphoma” not [“NHL” or “non”]	12	58.8	5:7	8.3	41.7	16.7	33.3
Lung	[“lung” and “origin”] or [“lung” and “primary”] or [“non” and “small” and “cell”] or [“lepidic”] or [“pulmonary” and “origin”] or [“bronchogenic”] or [“NSCLC”]	1695	69.7	819:876	3.9	25.9	33.7	36.5
Melanoma	“melanoma”	555	66.8	343:212	3.2	40.7	42.0	14.0
Mesothelioma	“mesothelioma”	77	71.6	55:22	3.9	37.7	42.9	15.6
Mycosis fungoides	“mf” or “mycosis” or “fungoides”	23	58.9	11:12	0	13.0	34.8	52.1
Neuroendocrine	“neuroendocrine” or “merkel” or “carcinoid”	268	67.2	150:118	2.6	62.3	28.4	6.7
Ovary	“ovary” or “ovarian”	256	62.3	0:256	2.0	61.3	33.2	3.5
Pancreas	“pancreas” or “pancreatic” or “ampulla”	286	65.0	153:133	2.8	54.6	31.8	10.8
Prostate	“prostate” and “prostatic”	193	72.4	193:0	3.1	63.2	23.8	9.8
Renal	“kidney” or “renal”	246	66.6	142:104	3.7	45.1	30.1	21.1
Sarcoma	“sarcoma”	140	63.8	69:71	2.1	40.7	23.6	33.6
Thymoma	“thymoma”	9	57.2	3:6	11.1	22.2	22.2	44.4
Thyroid	“thyroid”	68	61.3	18:50	4.4	49.0	20.6	26.5
Urothelial	“bladder” or “urothelial” or “transitional”	273	70.9	177:96	2.9	46.5	31.9	18.7
Vulva or vagina	“vulva” or “vagina”	85	64.4	0:85	2.3	44.7	31.8	21.2
Adenocarcinoma	“adenocarcinoma”	9575	69.1	4302:5273	3.2	33.7	34.5	28.6
Poorly differentiated adenocarcinoma	[“poorly” and “differentiated”] and “adenocarcinoma”	974	67.8	486:488	2.5	22.1	27.2	48.3
Moderately differentiated adenocarcinoma	[“moderately” and “differentiated”] and “adenocarcinoma”	676	69.1	304:372	2.5	36.8	38.6	22.0
Well-differentiated adenocarcinoma	[“well” and “differentiated”] and “adenocarcinoma”	278	71.5	114:164	4.7	49.6	38.1	7.6
Mucinous adenocarcinoma	“mucinous” and “adenocarcinoma”	226	69.7	111:115	3.5	51.3	34.1	11.1
Squamous cell carcinoma	“squamous” or “SCC” or “SCCA”	3102	70.5	1905:1197	2.3	26.4	40.8	30.5
Poorly differentiated squamous cell carcinoma	[“poorly” and “differentiated”] and “squamous” or “SCC” or “SCCA”	575	70.2	348:227	2.7	24.7	37.0	36.0
Moderately differentiated squamous cell carcinoma	[“moderately” and “differentiated”] and “squamous” or “SCC” or “SCCA”	508	71.6	312:196	0.6	24.2	44.3	30.9
Well-differentiated squamous cell carcinoma	[“well” and “differentiated”] and “squamous” or “SCC” or “SCCA”	71	68.6	42:29	1.4	16.9	54.9	26.8
Adenosquamous	“adenosquamous”	82	73.5	48:34	0	20.7	35.4	43.9
Metastatic	“metastatic”	3933	67.5	1944:1989	2.6	35.9	30.8	30.7
Pleural fluid	[“pleural” and “fluid”] or [“pleural” and effusion”]	2105	72.1	1025:1080	3.1	29.6	32.6	34.6
Pericardial fluid	“pericardial” and “fluid”	44	66.5	15:29	0	0	0	100
Bone	“bone”	2275	68.3	1246:1029	3.3	40.7	29.2	26.7
Metastases in brain	“brain” and “metastatic”	47	62.9	18:29	2.1	42.6	23.4	31.9
Adrenal	“adrenal”	549	67.7	307:242	3.6	27.5	33.9	35.0

^a^Tumor type was assessed using a natural language search of submitted information for site and submitted clinical history. All cases may not be represented from larger data set due to ambiguous or missing information in submitted site/history

#### Evaluation of 22C3/tumor proportion score expression in tumor types

Site- or type-specific tumors showed differences when compared with all combined cases. No expression was seen most often in the following: colon (67%), prostate (63%), neuroendocrine (62%), ovary (61%), and endometrial (55%). Highly expressed cases were most common in mycosis fungoides (52%), thymoma (44%), lung (37%), and sarcoma (34%).

#### Comparison of 22C3/tumor proportion score in well-, moderately and poorly differentiated adenocarcinoma and squamous cell carcinoma and adenosquamous carcinoma

Although only a subset of cases were captured using this search (*n* = 1928), we compared the overall results of adenocarcinoma identified as well- vs. moderately and poorly differentiated. We noted a statistically significant difference between poor- and well-differentiated cases (*p* = 0.02), although moderately differentiated cases were not significantly different from well- or poorly differentiated cases [Table 6a]. Furthermore, cases identified as mucinous adenocarcinomas were far more similar to well-differentiated as opposed to poorly differentiated adenocarcinomas (*p* = 0.91 compared with well-differentiated vs. *p* = 0.02 compared with poorly differentiated).

**Table 6a:** Kruskal–Wallis test followed by Dunn’s test for pairwise comparisons in adenocarcinoma/22C3/TPS

Comparisons	*P*
Moderate–Mucinous	0.07
Moderate–Poor	0.57
Mucinous–Poor	0.02*
Moderate–Well	0.09
Mucinous–Well	0.91
Poor–Well	0.02*

Similarly, we compared cases of squamous cell carcinoma identified as well- vs. moderately and poorly differentiated [Table 6b]. In contrast to adenocarcinoma, which showed greatest expression in poorly differentiated cases (75.5%), squamous cell carcinoma showed greatest expression in well-differentiated cases (81.7%). Poorly differentiated and moderately differentiated cases were found to be statistically significantly different from well-differentiated cases (*p* = 0.03 and *p* = 0.04, respectively). Adenosquamous carcinoma was statistically significantly different from poorly differentiated cases (*p* = 0.04) and trending toward significance from moderately differentiated cases (*p* = 0.05).

**Table 6b:** Kruskal–Wallis test followed by Dunn’s test for pairwise comparisons in squamous cell carcinoma/22C3/TPS

Comparisons	*P*
Adenosquamous–Moderate	0.05
Adenosquamous–Poor	0.04*
Moderate–Poor	0.91
Adenosquamous–Well	0.91
Moderate–Well	0.04*
Poor–Well	0.03*

#### Evaluation of 22C3/tumor proportion score expression in metastases

Expression pattern was evaluated in cases identified as metastatic (*n* = 3933). Other cases that were considered to be metastatic include the following: pleural fluid (*n* = 2105), pericardial fluid (*n* = 44), bone (*n* = 2259), adrenal (*n* = 549), and cases metastatic to brain (*n* = 47). Although each of these theoretically could have primary disease (such as, primary adrenal tumors), metastatic disease is far more likely. Furthermore, these groups may overlap somewhat, depending on the limitations of the submitted tumor history/site information. In cases identified as metastatic, the results were as follows: no expression 35.9% (*n* = 1411), expressed 30.8% (*n* = 1211), and highly expressed 30.7% (*n* = 1208) [Table 6].

**Fig. 3:**
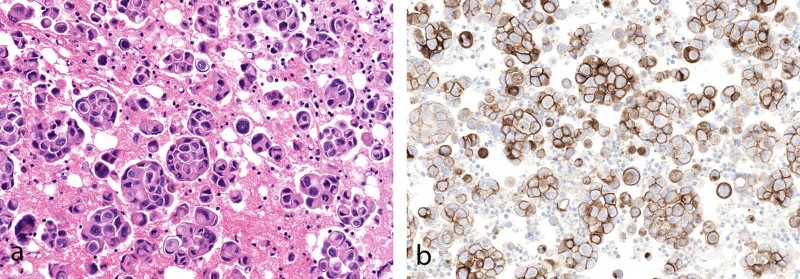
**a** Adenocarcinoma in pericardial fluid (H&E). **b** All cases analyzed showed tumor expression of PD-L1 22C3

Surprisingly, pericardial fluid had an exceedingly high rate of positivity (100% highly expressed) compared with pleural fluid, although only a relatively small number of pericardial cases were analyzed (*n* = 44) (Fig. 3).

Compared with all cases identified as metastatic, those identified as bone had a slightly higher quantity of not sufficient rate and only mildly increased numbers of no expression cases. The overall results do not show significant differences, suggesting that decalcification in bone specimens likely has no or little effect of PD-L1 results using 22C3.

#### Comparison of 22C3/tumor proportion score and combined positive score in esophageal/gastric cases

In many cases, tumors or adenocarcinomas of gastric, gastroesophageal, or gastroesophageal junction were submitted for evaluation for tumor proportion score rather than the combined positive score. However, as a result, this allows for a comparison of these cases using the combined positive score and tumor proportion scores, and indicates the degree of contribution to immune cell scoring for 22C3-stained cases. Scores for esophageal cancers were 49.2% with tumor proportion score vs. 85.8% with combined positive score (an increase of 36.6%). Score for gastric cancers were 50.3% with tumor proportion score vs. 83.6% with combined positive score (an increase of 33.3%).

### 28-8

The 28-8 antibody was evaluated in 4191 cases. The age range was 2–103 with an average age of 68 years. The male-to-female ratio was 53:47. Quantity not sufficient cases accounted for 4.7% (*n* = 197). Negative results were seen in 45.5% of cases (*n* = 1905) and positive results in 49.8% of cases (*n* = 2089) [Table 7]. High levels of expression ( > 40%) were identified in all subgroups analyzed: non-small cell lung cancer, urothelial carcinoma, melanoma, mesothelioma, adenocarcinoma, squamous cell carcinoma, and metastatic disease.

**Table 7:** Analysis of 28-8

Tumor type	Search parameter	Number of cases	Average age, years	M:F	QNS %	Negative (<1%)	Expressed ( > 1%)
All cases		4191	68	2229:1978	4.7	45.5	49.8
NSCLC	[“lung” and “origin”] or [“lung” and “primary”] or [“non” and “small” and “cell”] or [“lepidic”] or [“pulmonary” and “origin”] or [“bronchogenic”] or [“NSCLC”]	530	70	49:51	4.9	41.1	54.0
Melanoma	“melanoma”	507	67	300:207	5.3	49.5	45.2
Mesothelioma	“mesothelioma”	65	66	41:24	1.5	50.8	47.7
Urothelial	“bladder” or “urothelial” or “transitional”	29	70	20:9	0	48.3	51.7
Adenocarcinoma	“adenocarcinoma”	603	69	273:330	5.5	46.9	47.6
Squamous cell carcinoma	“squamous” or “SCC” or “SCCA”	261	71	154:107	4.2	31.8	64.0
Metastatic	“metastatic”	355	66	171:184	4.2	52.4	43.4

*NSCLC* non-small cell lung cancer

### SP142

The SP142 antibody was evaluated in 850 cases. The age range was 2–96 with an average age of 69 years. The male-to-female ratio was 48:52. Quantity not sufficient cases accounted for 6.2% (*n* = 53). Negative results were seen in 68.4% of cases and positive results in 25.4% of cases [Table 8].

**Table 8:** Analysis of SP142

Tumor type	Search parameter	Number of cases	Average age, years	M:F	QNS %	Negative ( < 1%)	Expressed ( > 1%)
All cases		850	69	441:409	6.2	68.4	25.4
Lung	“NSCLC”^a^	733	68	363:370	6.9	23.5	69.6
Breast	“breast” or “lobular” or [“ductal” not “pancreatic”]	49	52	0:49	8.1	79.6	12.2
Esophagus	“esophagus” or “esophageal” or “GEJ”	15	64	13:2	0	86.7	13.3
Adenocarcinoma	“adenocarcinoma”	101	66	45:56	10.9	72.3	16.8
SCC	“squamous” or “SCC” or “SCCA”	45	72	23:22	15.6	57.8	26.7
Metastatic	“metastatic”	58	64	28:30	6.9	82.8	10.3

^a^In contrast to other antibodies, NSCLC was indicated as a distinct parameter in database for SP142

### SP263

The SP263 antibody was evaluated in 70 cases. The age range was 2–94 with an average age of 64 years. The male-to-female ratio was 25:45. Quantity not sufficient cases accounted for 10% (*n* = 7). No expression seen in 62.9% of cases (*n* = 44) and positive results in 35.7% of cases (*n* = 19) [Table 9]. Cases of urothelial carcinoma (*n* = 21) were evaluated separately and showed positive results in 47.6%. No further subset analysis was performed because of the small number of cases of each type.

**Table 9:** Analysis of SP263

Tumor type*	Search parameter	Number of cases	Average age, years	M:F	QNS %	Negative ( < 1%)	Expressed ( > 1%)
All cases		70	64	25:45	10.0	62.9	27.1
Urothelial	“bladder” or “urothelial” or “transitional”	21	71	10:11	0	52.4	47.6

### Comparison of 22C3/tumor proportion score, 28-8, and SP142 in tumor types

As has been highlighted in other publications, SP142 had the lowest levels of positivity [Table 10]. This was true in all tumor types examined: lung, breast, esophagus, adenocarcinoma, squamous cell carcinoma, and metastatic disease.

**Table 10:** Comparison of PD-L1 expression in tumor types

Antibody	Overall positive	Adenocarcinoma positive	Squamous cell carcinoma positive	Metastases positive
22C3-TPS	63.2%	63.1%	71.3%	61.5%
22C3-CPS	79.0	80.0%	83.6%	68.3%
28-8	49.8%	47.6%	61.1%	43.4%
SP142	24.1%	16.8%	26.7%	10.3%

### Comparison with Keynote and Checkmate studies

We compared our results with those published previously in select Keynote and Checkmate studies [8, 12, 17–21]. These results are summarized in Table 11. In Keynote 59, evaluation of gastroesophageal carcinomas using combined positive score, we showed significant differences from the reported results (*χ*^2^ = 142, *p*- value < 0.00001) [Supplementary Materials 3]. We compared our results with Keynote 21, comparing expression in lung cancers for PD-L1, using our results for 22C3. In this case, our results were not statistically significant from those reported in Keynote 21 (*χ*^2^ = 5, *p*-value = 0.07) [Supplementary Materials 3]. We compared the results of Keynote 10, previously treated advanced non-small cell lung cancer, with our results for all lung cancers. Or results were significantly different than those of Keynote 10 (*χ*^2^ = 103, *p*-value < 0.0001) [Supplementary Materials 3].

**Table 11:** Comparison of current study to results of Checkmate and Keynote studies

Study	Description	Current study group/antibody–scoring	*χ* ^2^	*P*-value
Keynote 59	Gastroesophageal carcinoma	Gastroesophageal carcinomas/22C3-CPS	142	< 0.00001
Keynote 21	Lung cancer	Lung cancer/22C3-TPS	5	0.07
Keynote 10	Previously treated NSCLC	Lung cancer/22C3-TPS	103	< 0.0001
Checkmate 275	Urothelial cancer	Urothelial cancer/28-8-tumor cell expression	0.6	0.81
Checkmate 57	Metastatic lung cancer	Lung cancer/22C3-TPS	0.7	0.40
	Metastatic lung cancer	Metastases/22C3-TPS	5.9	0.01
Checkmate 141	Recurrent or metastatic squamous cell carcinoma	Squamous cell carcinoma/28-8 tumor cells	4.3	0.04
Checkmate 67	Melanoma	Melanoma/28-8 tumor cells	0.7	0.39
Checkmate 238	Adjuvant therapy for melanoma	Melanoma/28-8 tumor cells	24	< 0.00001

*CPS* combined positive score; *TPS* tumor proportion score

In urothelial cancer, using the 28-8 antibody, our results did not differ significantly from Checkmate 275 (*χ*^2^ = 0.6, *p*-value = 0.81) [Supplementary Materials 3]. Likewise, no significant difference was identified in comparison with Checkmate 57, metastatic small cell lung cancer, compared with all lung cancers in the study (*χ*^2^ = 0.70, *p*-value = 0.40) [Supplementary Materials 3]. However, when compared with all metastases in our study, the results were statistically different (*χ*^2^ = 5.9, *p*-value = 0.01) [Supplementary Materials 3]. In Checkmate 141, recurrent or metastatic squamous cell carcinoma was compared with all squamous cell carcinomas in the current study evaluated by 28-8. These results were statistically different (*χ*^2^ = 4.3, *p*-value = 0.04) [Supplementary Materials 3]. In Checkmate 67, results of 28-8 expression in melanoma were compared and no statistically significant difference was identified (*χ*^2^ = 0.7, *p*-value = 0.39) [Supplementary Materials 3]. However, different levels of expression were seen when comparing Checkmate 238 (adjuvant therapy for melanoma). In these cases, evaluated by 28-8 our results showed a *χ*^2^ = 24, *p*-value < 0.00001) [Supplementary Materials 3].

### Practical observations in PD-L1 interpretation

**Fig. 4:**
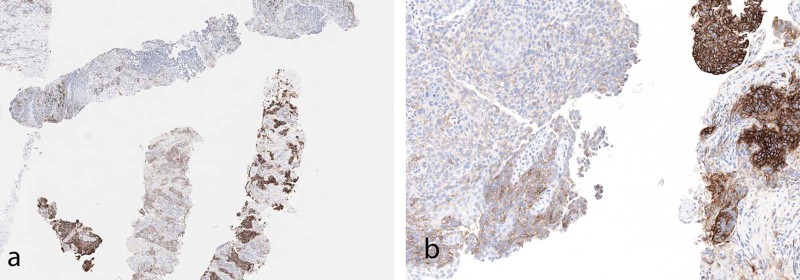
Examples (**a**, **b**) of intratumoral variation in PD-L1 staining intensity from 0 to 3 + (22C3)

**Fig. 5:**
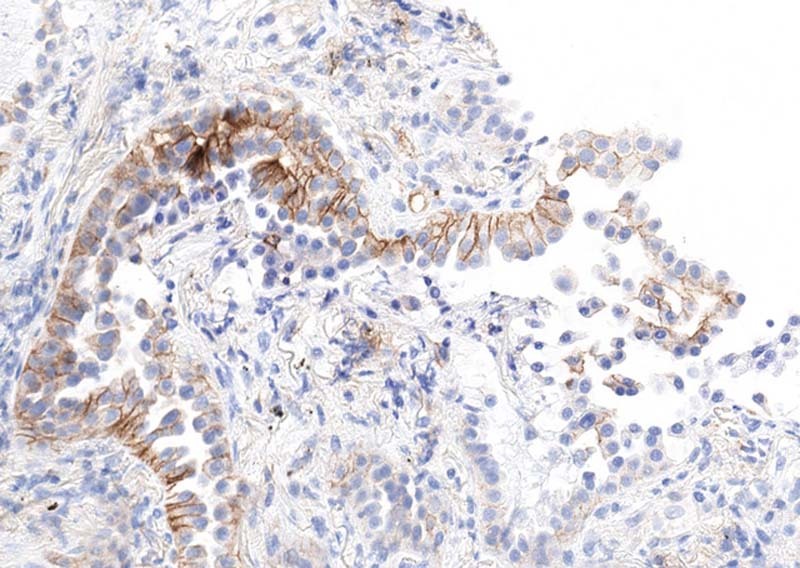
Partial membranous PD-L1 staining (no staining at apical surface), which is still considered positive

**Fig. 6:**
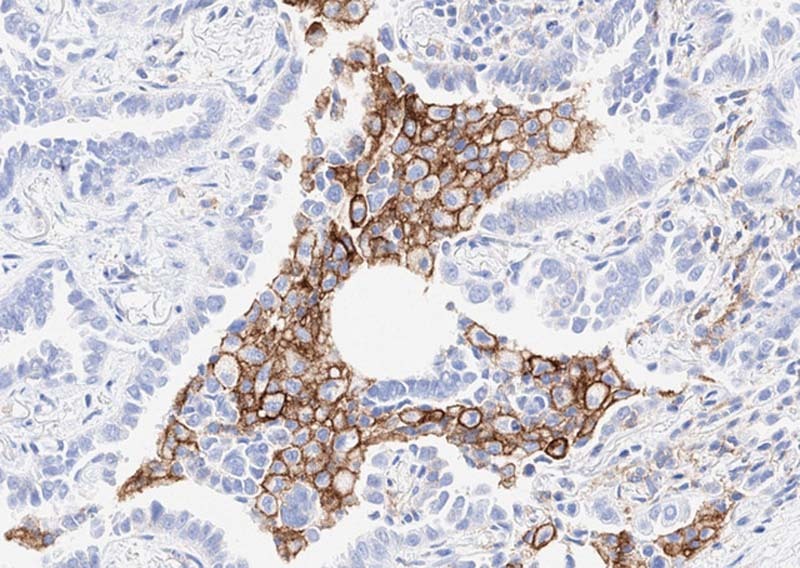
Strong positive PD-L1 staining (3 + ) in luminal macrophages with no staining in tumor glandular cells (22C3)

Interpretation should be performed according to specific instructions for each antibody and indication. Intensity of staining can vary significantly within a single case (Fig. 4). Furthermore, positive staining in tumor cells should be membranous, but does not have to encompass the entire membrane (Fig. 5). Staining of the apical surfaces only within glands is not considered a positive result. Occasionally, macrophages within gland lumens are strongly positive, with no staining in tumor cells (Fig. 6). This is not generally considered as a positive result.

Cytologic specimens can be especially challenging to interpret, especially when there is cytologic atypia of positive histiocytes, and tumor cells or clusters are of a comparable size. This is especially true in cytologic specimens with large numbers of histiocytes. As in all cytology specimens, when tumor cells are rare, interpretation and scoring can be a challenge. Rarely, if tumors cells are positive, with negative staining in immune cells, then their appearance can be quite easy to detect on the PD-L1 stain.

**Fig. 7:**
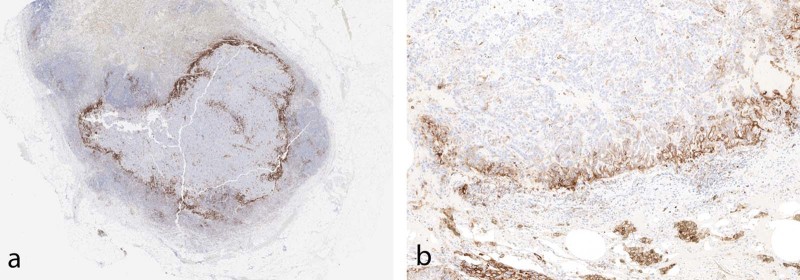
**a** Low-magnification image of PD-L1 staining in tumor metastatic to lymph node. At the interface of tumor and non-tumor, there is intense staining in immune cells. **b** High magnification in tumor showing strong staining at the tumor interface, with immune cells positive and tumor cells at the interface positive. Tumor cells further from the interface are not positive (22C3)

In occasional cases, positivity of tumor cells at an interface of tumor and stroma or histiocytes/lymphocytes can be seen with no significant staining within the more central portions of the tumor (Fig. 7). This edge effect is likely a result of direct interaction between the tumor cell antigens and upregulation of PD-L1 expression by adjacent immune cells. This should be distinguished from the frequently seen edge artifact identified in many immunohistochemical stains.

#### Normal staining and artifacts

**Fig. 8:**
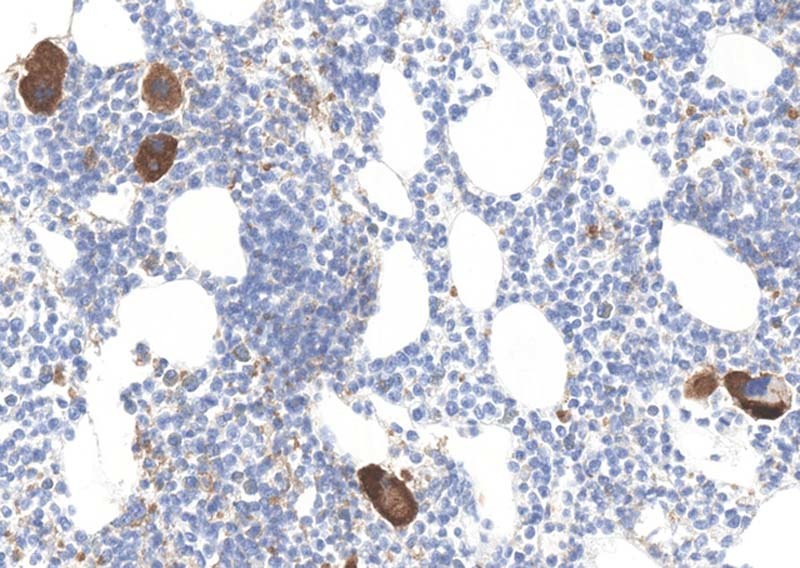
Strong cytoplasmic staining of megakaryocytes for PD-L1 (22C3). Staining is noted in all antibodies

As mentioned previously, expression of PD-L1 can be seen in many histiocytes/macrophages in various body sites. Other cells that are usually or always positive for PD-L1 staining include perineurial cells, nerve fibers, plasma cells, follicular dendritic cells, mast cells, and megakaryocytes (Fig. 8).

**Fig. 9:**
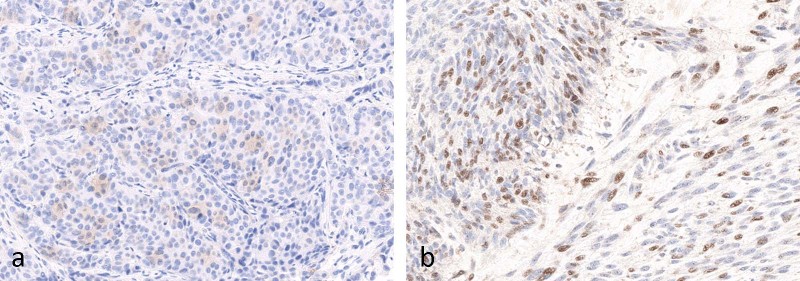
**a** Faint cytoplasmic staining for PD-L1 in breast carcinoma. This is not considered to be true positive staining (22C3). **b** Nuclear staining for PD-L1. This is not considered to represent positive staining in any scoring systems (22C3)

Bacteria and acellular debris may have significant positivity and are ignored for stain interpretation. Furthermore, as platelets express PD-L1, their aggregation in debris or tissue may impart positivity. Although incomplete membrane staining is considered positive, granular cytoplasmic staining in tumor cells is not considered positive in any of the scoring systems (Fig. 9). Rarely, nuclear staining may be identified but is not considered positive in any scoring systems (Fig. 9).

**Fig. 10:**
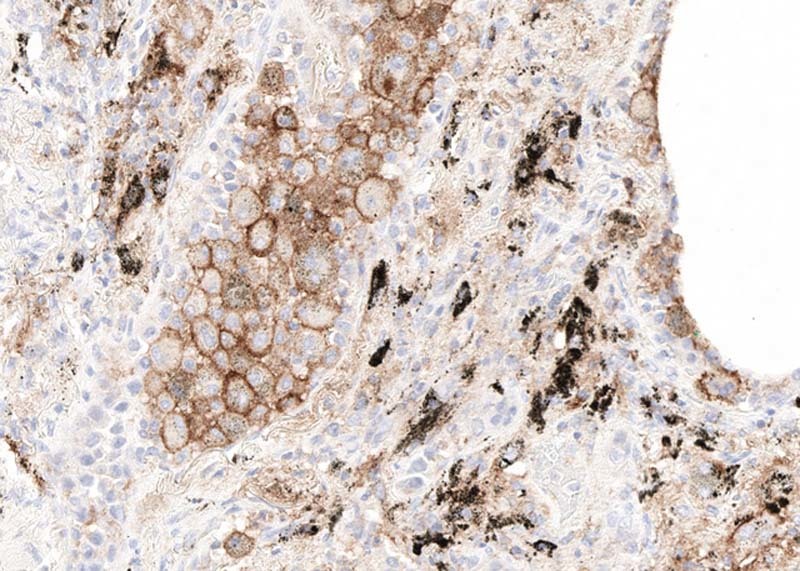
Anthracotic pigment (black) and strong PD-L1 staining in macrophages with no staining in tumor cells (22C3)

Rarely, pigment can complicate stain interpretation. Melanin pigment in primary or metastatic melanomas, anthracotic pigment (typically in the lung and hilar lymph nodes), tattoo pigment (lymph nodes), or extensive hemosiderin deposition need to be carefully excluded for PD-L1 interpretation (Fig. 10). As in all cases, careful assessment of immunohistochemical stains, positive and negative controls, as well as comparison with hematoxylin and eosin-stained specimens can minimize this difficulty.

## Discussion

PD-L1 expression in tumors and, in some cases, immune cells, evaluated by immunohistochemical staining, is currently a highly used test in conjunction with anti-PD1 and anti-PD-L1 therapies. Good-to-excellent reliability of scoring of PD-L1 expression on tumor cells has been demonstrated, although immune cell scoring has a lower reliability [15, 22]. We present the results of a large number of tests in a broad range of tumor types and using a variety of available stains.

We showed significantly higher expression in gastroesophageal carcinomas compared with the original Keynote 59 study [Table 11]. Results from initial studies could be affected by case selection with biases toward advanced stage disease, higher pathologic grade, or those cases with a marked immune reaction. In addition, we found significant differences from reported results of Keynote 10 (previously treated non-small cell lung cancer), Checkmate 141 (recurrent or metastatic squamous cell carcinoma), and Checkmate 238 (adjuvant therapy for melanoma). The comparisons of the current study results with those from the studies are not exact. For example, we compared Checkmate 141 with “all” squamous cell carcinoma results and “all” melanoma results in Checkmate 238, although the results compared are only with stated stain results and not keyed to outcomes. This is highlighted by comparison with the result of Checkmate 57 (metastatic lung cancer). When compared with the current results for lung cancers, the results were not significant (*p* = 0.4); however, when compared with results of all metastases, the results were significant (*p* = 0.01). We did not show significant differences to those published in Checkmate 275 (urothelial carcinoma), Keynote 21 (lung cancer), or Checkmate 67 (melanoma). Our study parallels that of Rimm et al [23], which shows that SP142 had considerably lower reactivity than other antibodies [Table 10].

Our data suggest that there are fewer negatives and higher overall expression in poorly differentiated adenocarcinomas vs. those identified as well-differentiated adenocarcinomas (within the largest data group, 22C3-TPS) (poorly differentiated 75.5% expressed, moderately differentiated 60.6% expressed, and well-differentiated 45.7% expressed). However, statistical comparisons show significance only between poorly differentiated adenocarcinomas from well-differentiated adenocarcinomas (*p* = 0.02) [Table 6a]. Comparisons of moderate- to poor- and moderate- to well-differentiated adenocarcinomas were not significant (*p* = 0.57 and 0.09, respectively). This would support the general hypothesis that more neoantigens generate more potent expression of the immune checkpoint markers [24].

In parallel, mucinous adenocarcinomas were also statistically different from poorly differentiated adenocarcinomas (*p* = 0.02), but not compared with moderate (*p* = 0.07) or well (*p* = 0.91) differentiated adenocarcinomas. In spite of a tendency to be histologically poorly differentiated, mucinous adenocarcinomas appear to have similar reactivity to that of well-differentiated adenocarcinomas. A possible explanation is that the tumor antigens are not exposed to the immune response, as they are masked by mucus or that there are generally fewer tumor-infiltrating lymphocytes [25].

In contrast to adenocarcinomas, in the 22C3/tumor proportion score group, squamous cell carcinomas have increasing positivity, as there is greater differentiation (poorly differentiated 73.0%, moderately differentiated 75.2%, and well-differentiated 81.7%). There are statistically significant differences between well- and moderately differentiated squamous cell carcinoma (*p* = 0.04) and well- and poorly differentiated ones (*p* = 0.03) [Table 6b]. Adenosquamous carcinoma is significantly different from poorly differentiated squamous cell carcinoma (*p* = 0.04), borders on significance from moderately differentiated (*p* = 0.05) and is not significantly different from well-differentiated ones (*p* = 0.91).

There is no obvious answer as to the differences identified in pericardial tumor expression (100% strong expression) vs. that of pleural fluid (30% no expression, 33% expression, and 35% strong expression). However, pericardial effusions are far more rare than pleural effusions and involvement of the pericardial space may have a more robust or vigilant immune response, whereas the pleural fluid may be somewhat more permissive to the immunologic challenges of tumor involvement.

The data presented is a reflection of the ordering patterns of pathologists and the requests of oncologists. These may reflect “off-label” uses, such as requesting combined positive scores on samples that do not have a current indication and do not have supportive research for their use. Conversely, other orders do not request the appropriate antibody or scoring system for the intended drug being used. Although these may represent a practical approach to the “information overload” associated with myriad available antibodies and scoring systems, it would appear that the burden of education in this areas lies most heavily on the drug manufacturers and the producers of the antibodies.

Our data show that there are large numbers of tests being performed in tumors to assess PD-L1 expression. The efficacy of this testing in a coordinated manner, with the use of best performing antibodies, rather than those approved for companion or complementary diagnostic testing, may provide a better and more consistent understanding of the tumor and immune cell expression of PD-L1. This data raises many interesting questions about expression patterns of anti-PD-L1 antibodies in tumors and immune cells. It is likely to be that future research will be able to identify subsets of results that are able to better predict the most efficacious responses of the anti-PD1/PD-L1 therapies.

## Supplementary information

Supplemental materials 1

Supplemental material 2

Supplemental materials 3
