# Supplementary material for: Immunohistochemical detection of PD-L1 among diverse human neoplasms in a reference laboratory: observations based upon 62,896 cases
Source: Mod Pathol. 2019 Feb 13;32(7):929–42. doi: 10.1038/s41379-019-0210-3 (PMC6760643; doi:10.1038/s41379-019-0210-3)
Supplement: Supplementary file 1 — Supplemental materials 1 [file 41379_2019_210_MOESM1_ESM.docx]

Supplemental table 1. Quality assurance testing for PD-L1 22C3 TPS

| Month | Expressed | High expression | No expression | QNS | % positive |
| --- | --- | --- | --- | --- | --- |
| 1 | 1101 | 868 | 1007 | 88 | 66.2 |
| 2 | 989 | 897 | 1047 | 94 | 64.3 |
| 3 | 1140 | 1034 | 1207 | 116 | 64.3 |
| 4 | 1026 | 819 | 1042 | 84 | 63.9 |
| 5 | 1082 | 981 | 1140 | 85 | 64.4 |
| 6 | 918 | 908 | 1125 | 96 | 61.9 |

% positive excludes: QNS, indeterminate, clinical trials.

Supplemental table 2. Quality assurance testing for PD-L1 22C3 CPS

| Month | High expression | No expression | QNS | % positive |
| --- | --- | --- | --- | --- |
| 1 | 241 | 39 | 8 | 86.1 |
| 2 | 244 | 43 | 3 | 85.0 |
| 3 | 247 | 70 | 5 | 77.9 |
| 4 | 240 | 43 | 8 | 84.8 |
| 5 | 265 | 53 | 5 | 83.3 |
| 6 | 209 | 48 | 7 | 81.3 |
