# Supplementary material for: Immunohistochemical detection of PD-L1 among diverse human neoplasms in a reference laboratory: observations based upon 62,896 cases
Source: Mod Pathol. 2019 Feb 13;32(7):929–42. doi: 10.1038/s41379-019-0210-3 (PMC6760643; doi:10.1038/s41379-019-0210-3)
Supplement: Supplementary file 3 — Supplemental materials 3 [file 41379_2019_210_MOESM3_ESM.docx]

Supplemental material:

1) Comparison of adenocarcinoma grade (TPS score 22C3): from table 6

|  | Negative (<1) | Expressed (1-49%) | Highly expressed (>50) | Sum |
| --- | --- | --- | --- | --- |
| All adenocarcinoma | 3227 | 3303 | 2738 | 9268 |
| Poorly differentiated | 215 | 265 | 470 | 950 |
| Moderately differentiated | 249 | 261 | 149 | 659 |
| Well differentiated | 138 | 106 | 21 | 265 |
|  | 3829 | 3935 | 3378 |  |
| Mucinous adenocarcinoma | 116 | 77 | 25 | 226 |

Kruskal-Wallis test was run (P-value 0.03) followed by Dunn’s test for pairwise comparisons. Data analyzed in R version 3.5.

Adeno(P=0.03)

| Comparisons | P |
| --- | --- |
| Moderate - Mucinous | 0.07 |
| Moderate - Poor | 0.57 |
| Mucinous - Poor | 0.02* |
| Moderate - Well | 0.09 |
| Mucinous - Well | 0.91 |
| Poor – Well | 0.02* |

2) Comparison of squamous cell carcinoma grade

|  | Negative (<1) | Expressed (1-49%) | Highly expressed (>50) | Sum |
| --- | --- | --- | --- | --- |
| All squamous cell carcinoma | 819 | 1266 | 946 | 3102 |
| Poorly differentiated | 142 | 213 | 207 | 575 |
| Moderately differentiated | 123 | 225 | 157 | 508 |
| Well differentiated | 12 | 39 | 19 | 71 |
| Adenosquamous | 17 | 29 | 36 | 82 |
|  |  |  |  |  |

Squamous ( P= 0.04)

| Comparisons | P |
| --- | --- |
| Adenosquamous – Moderate | 0.05 |
| Adenosquamous – Poor | 0.04* |
| Moderate – Poor | 0.91 |
| Adenosquamous – Well | 0.91 |
| Moderate – Well | 0.04* |
| Poor – Well | 0.03* |

Antibody types and scores:

| Antibody | N | Overall positive | Adenocarcinoma positive | Squamous cell carcinoma positive | Metastases positive |
| --- | --- | --- | --- | --- | --- |
| 22C3 TPS | 52585 | 63.2% | 63.1% | 71.3% | 61.5% |
| 22C3 CPS | 2623 | 79.0% | 80.0% | 83.6% | 68.3% |
| 28-8 | 4191 | 49.8% | 47.6% | 61.1% | 43.4% |
| SP142 | 850 | 24.1% | 16.8% | 26.7% | 10.3% |

Positivity rates of antibodies across tumor types (P < 0.01)

| Comparisons | P |
| --- | --- |
| 22C3_CPS - 22C3_TPS | 0.3 |
| 22C3_CPS - 28-8 | 0.02* |
| 22C3_TPS - 28-8 | 0.21 |
| 22C3_CPS - SP142 | <0.01* |
| 22C3_TPS - SP142 | 0.01* |
| 28-8 - SP142 | 0.23 |

Correlation of positivity of 22C3 and 28-8 in lung cancer has also been observed in an earlier study (Batenchuk C, et al. J Clin Pathol . doi:10.1136/jclinpath-2018-205362)

Positivity across adenocarcinoma types as measured by antibodies (P-value 0.78, no significance)

| Comparisons | P |
| --- | --- |
| Adenocarcinoma - Metastases | 0.66 |
| Adenocarcinoma - Overall | 0.88 |
| Metastases - Overall | 0.55 |
| Adenocarcinoma - Squamous | 0.55 |
| Metastases - Squamous | 0.3 |
| Overall - Squamous | 0.66 |
