# Supplementary material for: Immunohistochemical detection of PD-L1 among diverse human neoplasms in a reference laboratory: observations based upon 62,896 cases
Source: Mod Pathol. 2019 Feb 13;32(7):929–42. doi: 10.1038/s41379-019-0210-3 (PMC6760643; doi:10.1038/s41379-019-0210-3)
Supplement: Supplementary file 3 [file 41379_2019_210_MOESM2_ESM.docx]

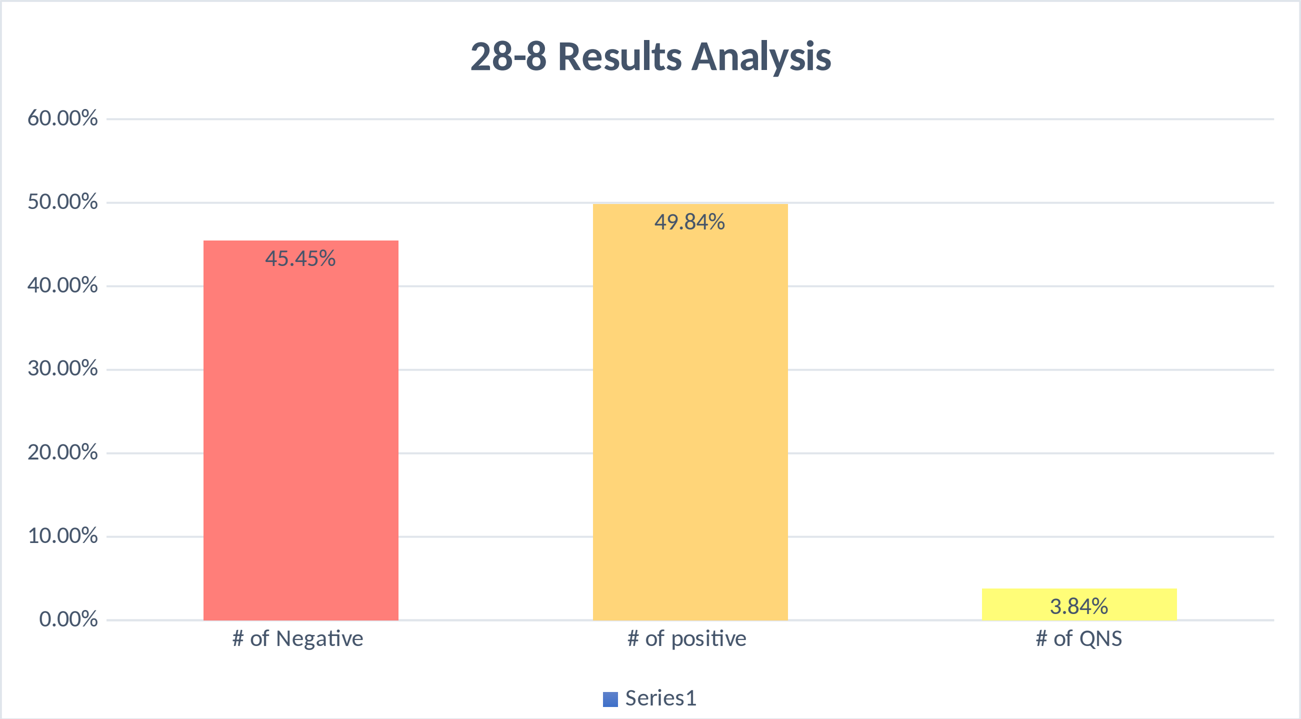
28-8 Results Analysis
60.00%
50.00%
40.00%
30.00%
20.00%
10.00%
0.00%
45.45%
49.84%
3.84%
# of Negative
# of positive
# of QNS
Series1

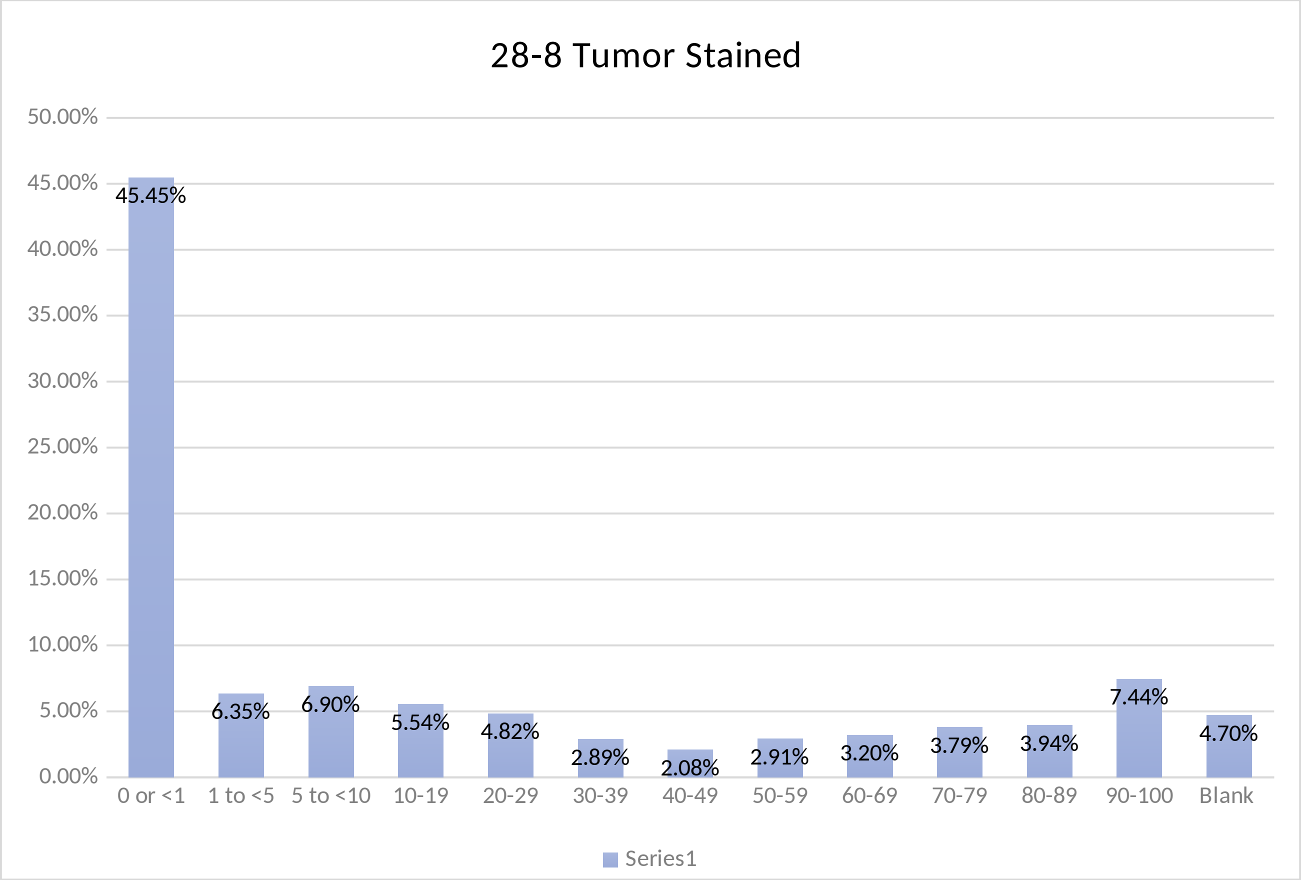
28-8 Tumor Stained
50.00%
45.00%
40.00%
35.00%
30.00%
25.00%
20.00%
15.00%
10.00%
5.00%
0.00%
45.45%
6.35%
6.90%
5.54%
4.82%
2.89%
2.08%
2.91%
3.20%
3.79%
3.94%
7.44%
4.70%
0 or <1
1 to <5
5 to <10
10-19
20-29
30-39
40-49
50-59
60-69
70-79
80-89
90-100
Blank
Series1

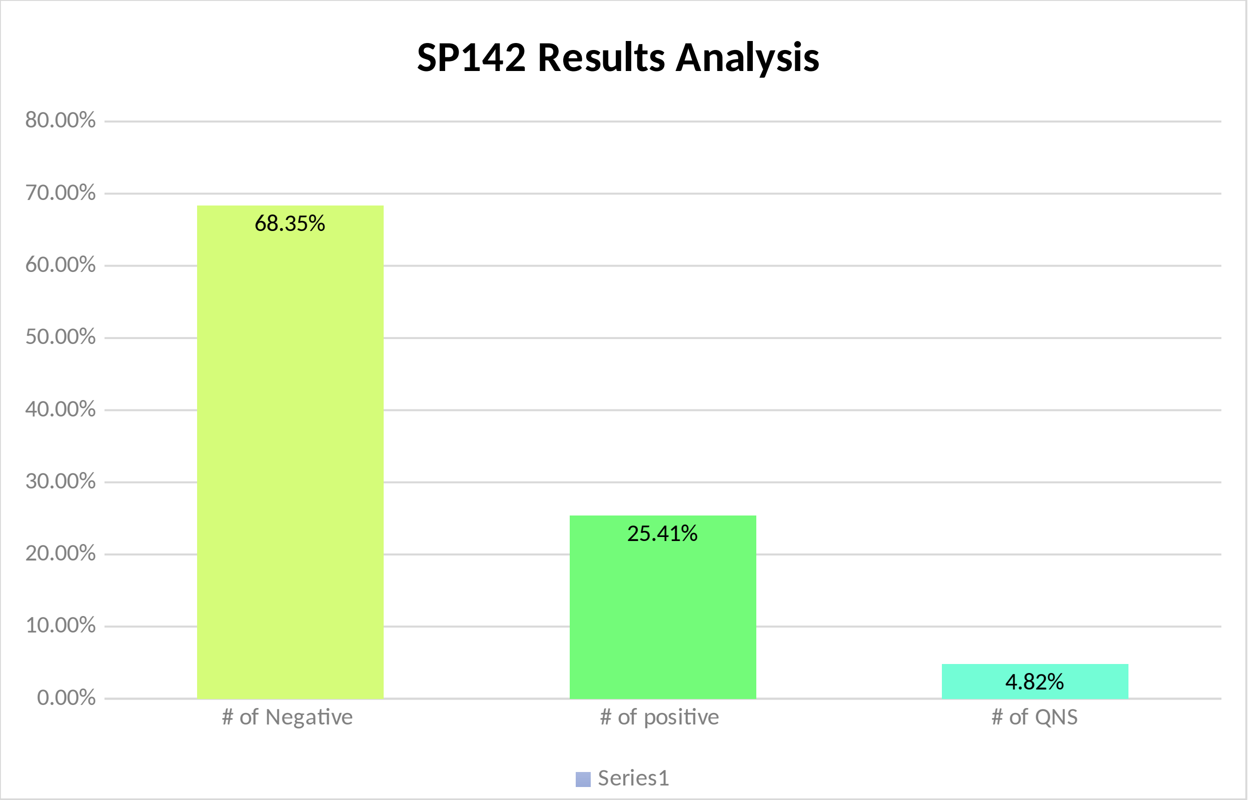
SP142 Results Analysis
80.00%
70.00%
60.00%
50.00%
40.00%
30.00%
20.00%
10.00%
0.00%
68.35%
25.41%
4.82%
# of Negative
# of positive
# of QNS
Series1

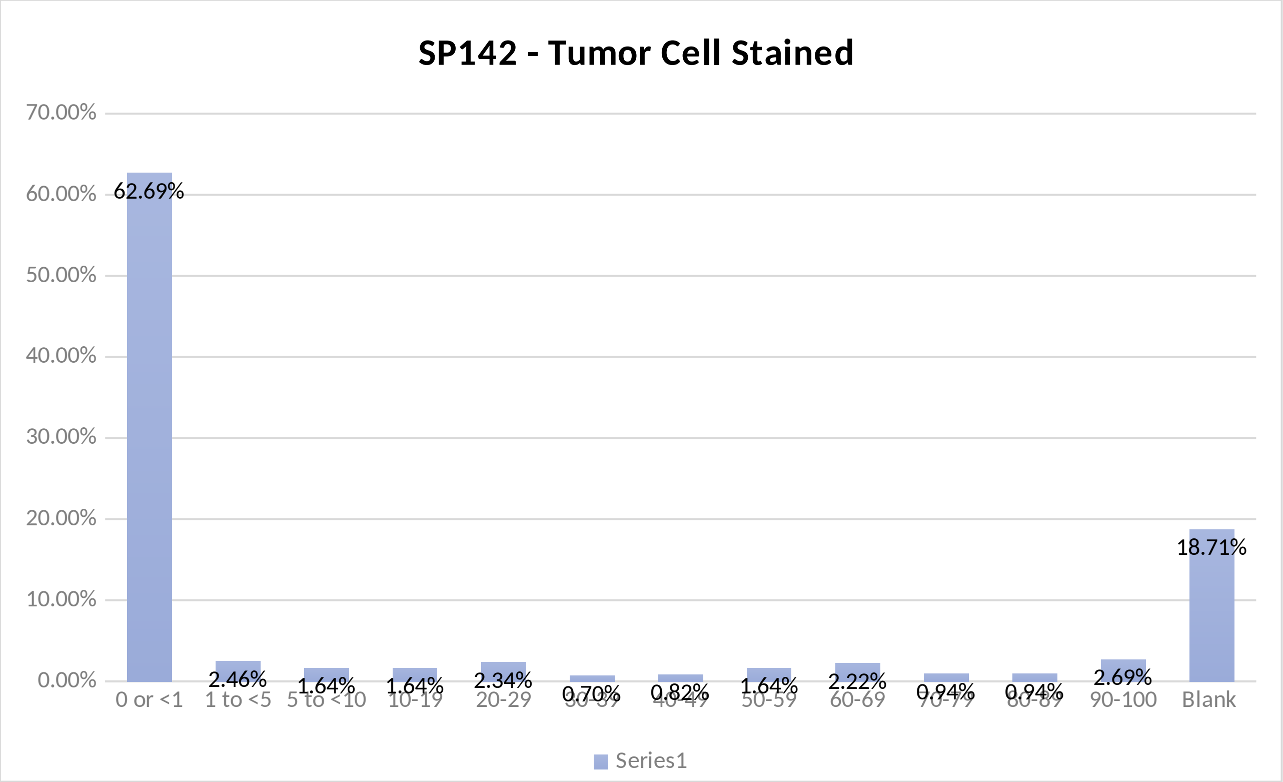
SP142 - Tumor Cell Stained
70.00%
60.00%
50.00%
40.00%
30.00%
20.00%
10.00%
0.00%
62.69%
2.46%
1.64%
1.64%
2.34%
0.70%
0.82%
1.64%
2.22%
0.94%
0.94%
2.69%
18.71%
0 or <1
1 to <5
5 to <10
10-19
20-29
50-59
60-69
90-100
Blank
Series1

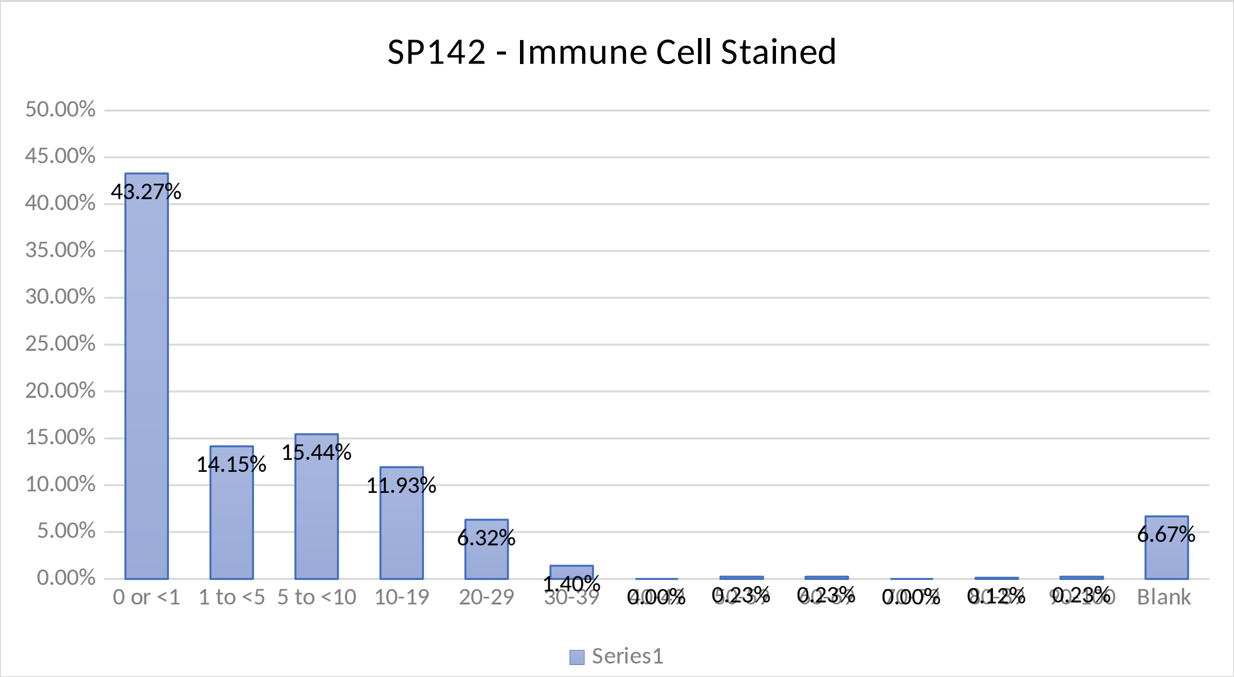
SP142 - Immune Cell Stained
50.00%
45.00%
40.00%
35.00%
30.00%
25.00%
20.00%
15.00%
10.00%
5.00%
0.00%
43.27%
14.15%
15.44%
11.93%
6.32%
1.40%
0.00%
0.23%
0.23%
0.00%
0.12%
0.23%
6.67%
0 or <1
1 to <5
5 to <10
10-19
20-29
30-39
Blank
Series1

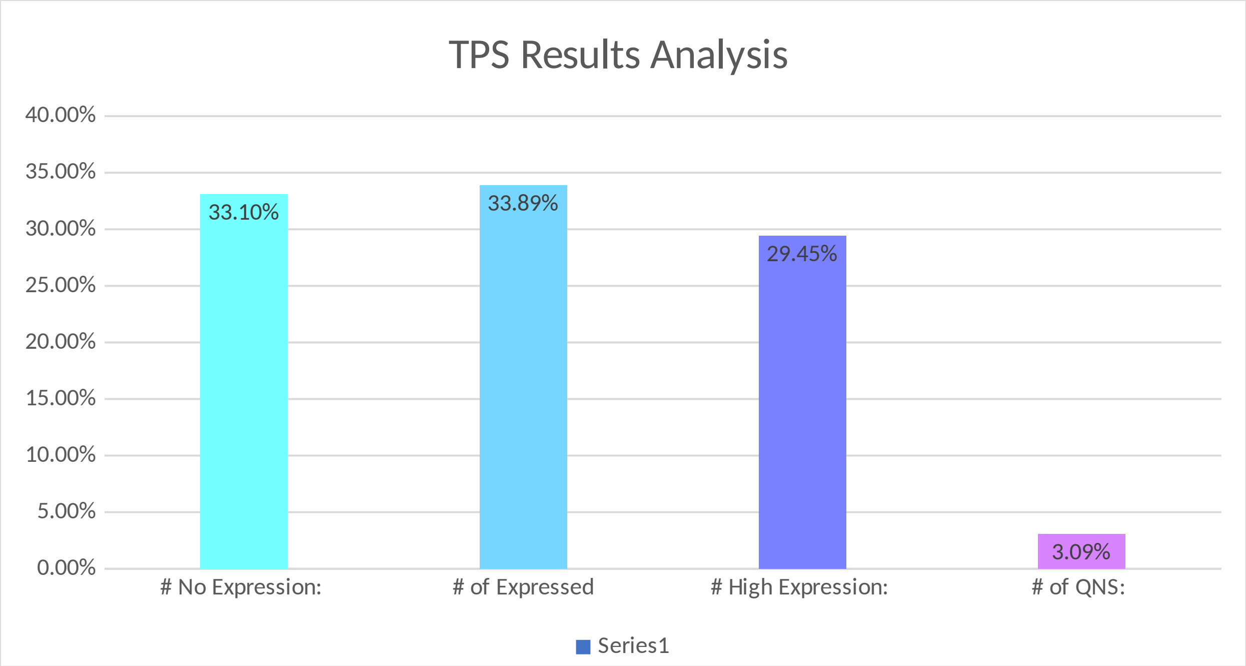

TPS Results Analysis
40.00%
35.00%
30.00%
25.00%
20.00%
15.00%
10.00%
5.00%
0.00%
33.10%
33.89%
29.45%
3.09%
No Expression:
of Expressed
High Expression:
of QNS:
Series1

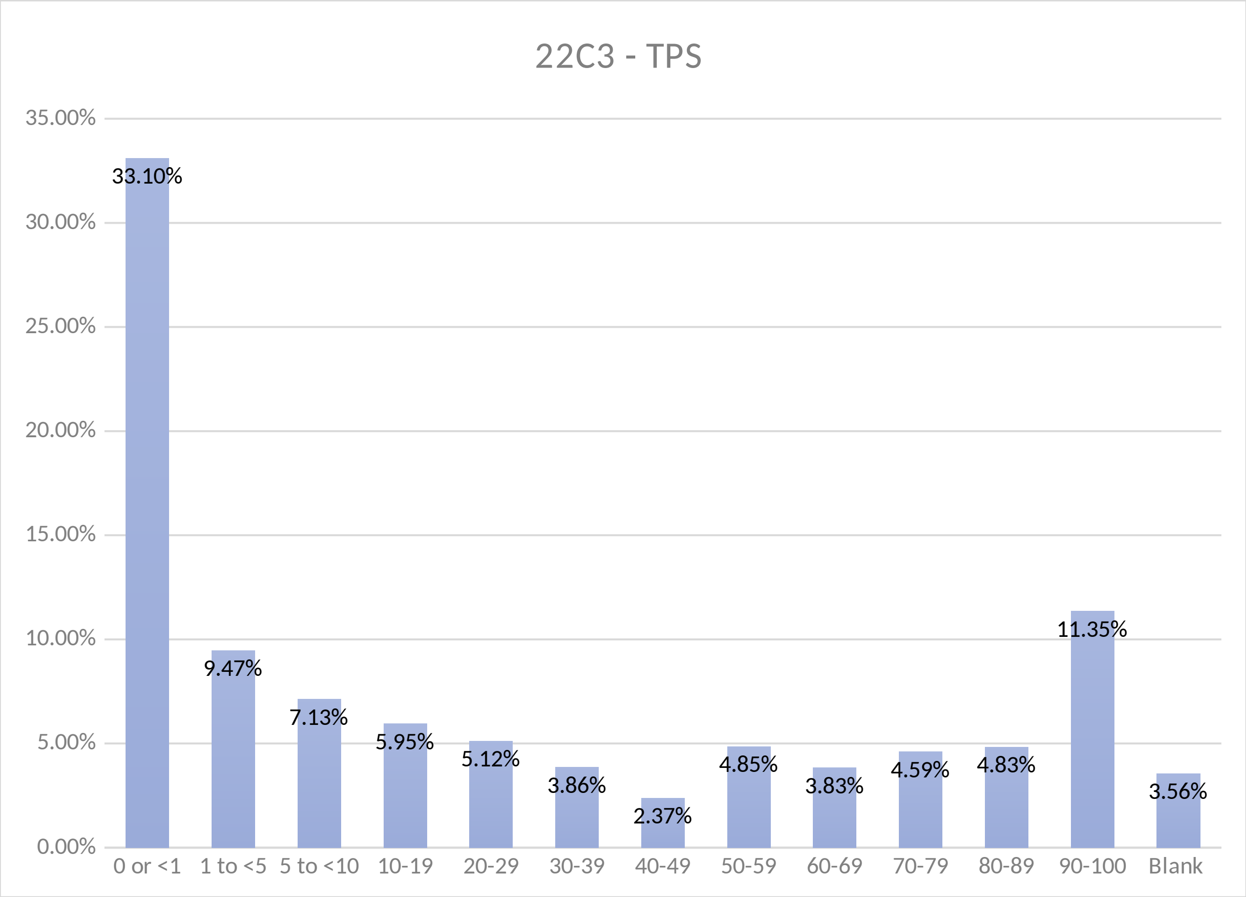

22C3 - TPS
35.00%
30.00%
25.00%
20.00%
15.00%
10.00%
5.00%
0.00%
33.10%
9.47%
7.13%
5.95%
5.12%
3.86%
2.37%
4.85%
3.83%
4.59%
4.83%
11.35%
3.56%
0 or <1
1 to <5
5 to <10
10-19
20-29
30-39
40-49
50-59
60-69
70-79
80-89
90-100
Blank

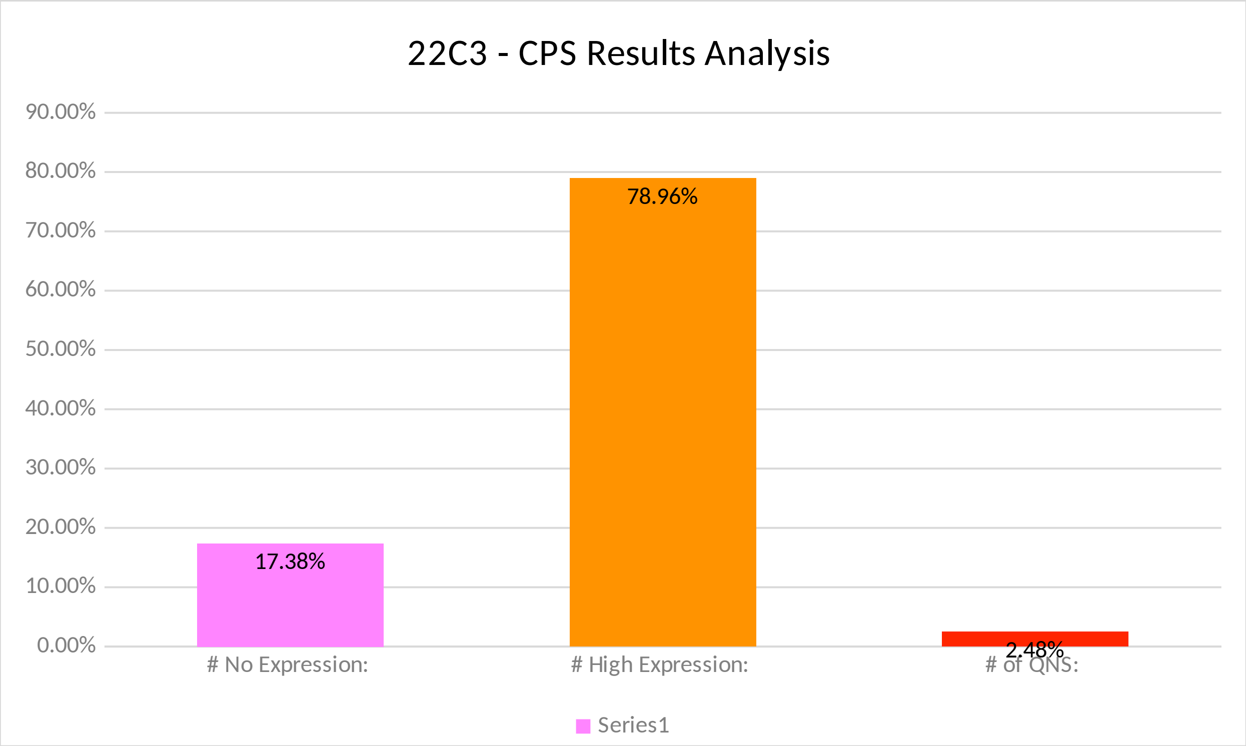
22C3 - CPS Results Analysis
90.00%
80.00%
70.00%
60.00%
50.00%
40.00%
30.00%
20.00%
10.00%
0.00%
17.38%
78.96%
2.48%
No Expression:
High Expression:
of QNS:
Series1

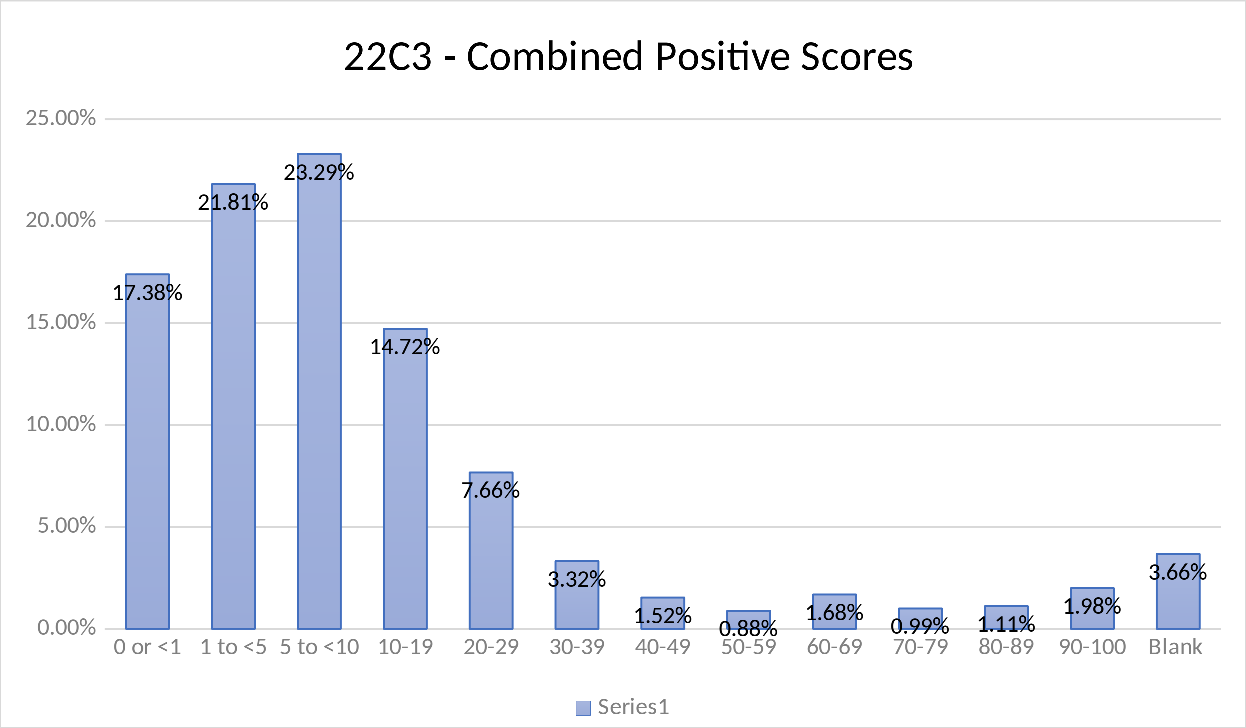
22C3 - Combined Positive Scores
25.00%
20.00%
15.00%
10.00%
5.00%
0.00%
17.38%
21.81%
23.29%
14.72%
7.66%
3.32%
1.52%
0.88%
1.68%
0.99%
1.11%
1.98%
3.66%
0 or <1
1 to <5
5 to <10
10-19
20-29
30-39
40-49
50-59
60-69
70-79
80-89
90-100
Blank
Series1

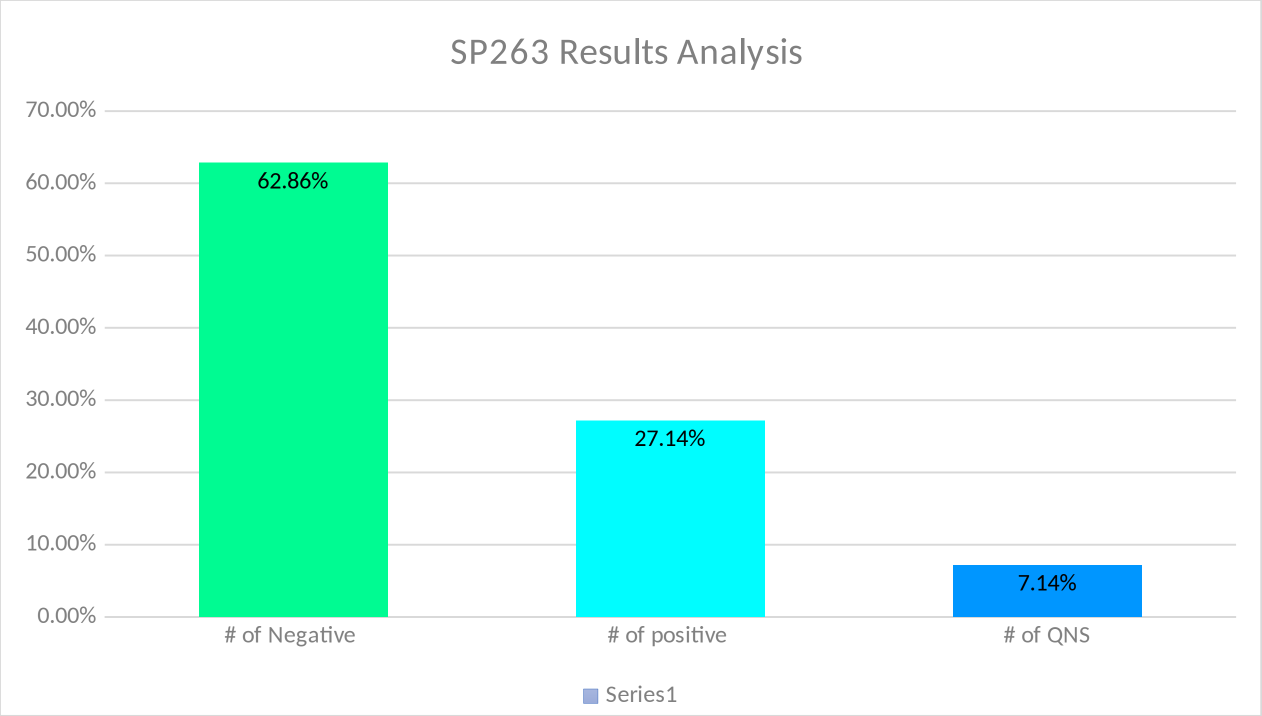
SP263 Results Analysis
70.00%
60.00%
50.00%
40.00%
30.00%
20.00%
10.00%
0.00%
62.86%
27.14%
7.14%
# of Negative
# of positive
# of QNS
Series1

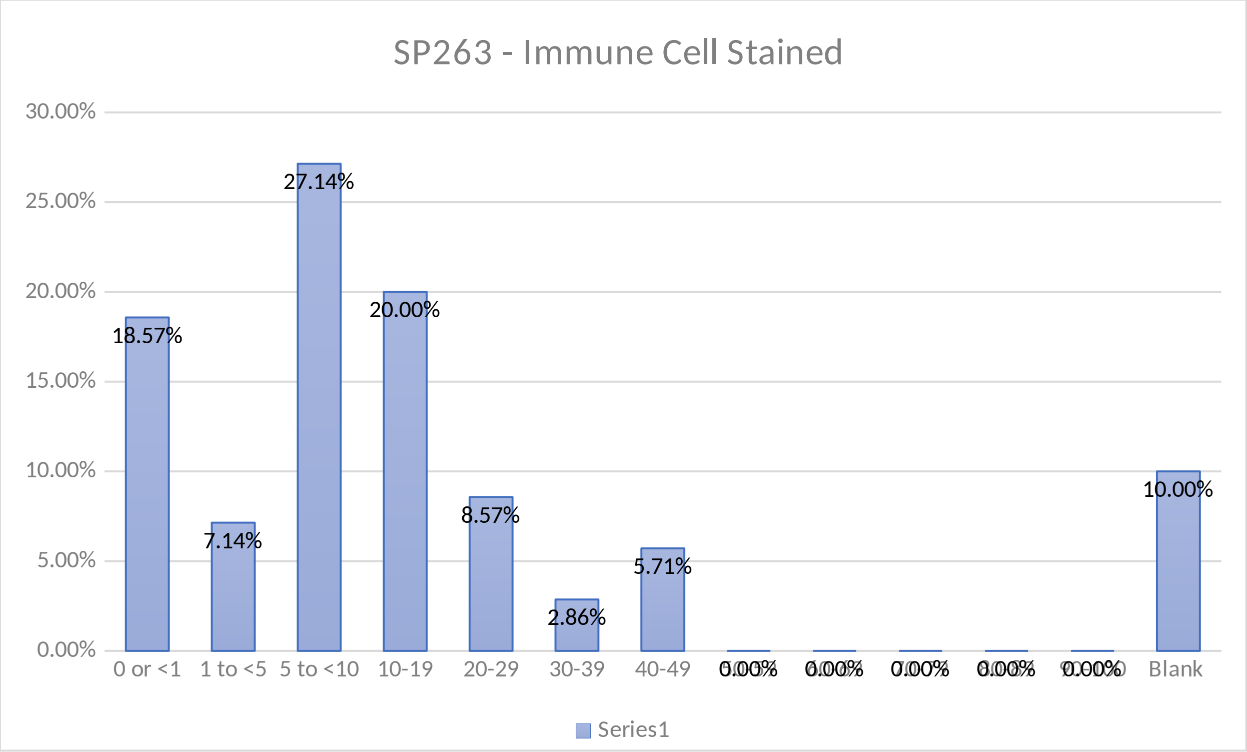
SP263 - Immune Cell Stained
30.00%
25.00%
20.00%
15.00%
10.00%
5.00%
0.00%
18.57%
7.14%
27.14%
20.00%
8.57%
2.86%
5.71%
0.00%
0.00%
0.00%
0.00%
0.00%
10.00%
0 or <1
1 to <5
5 to <10
10-19
20-29
30-39
40-49
Blank
Series1

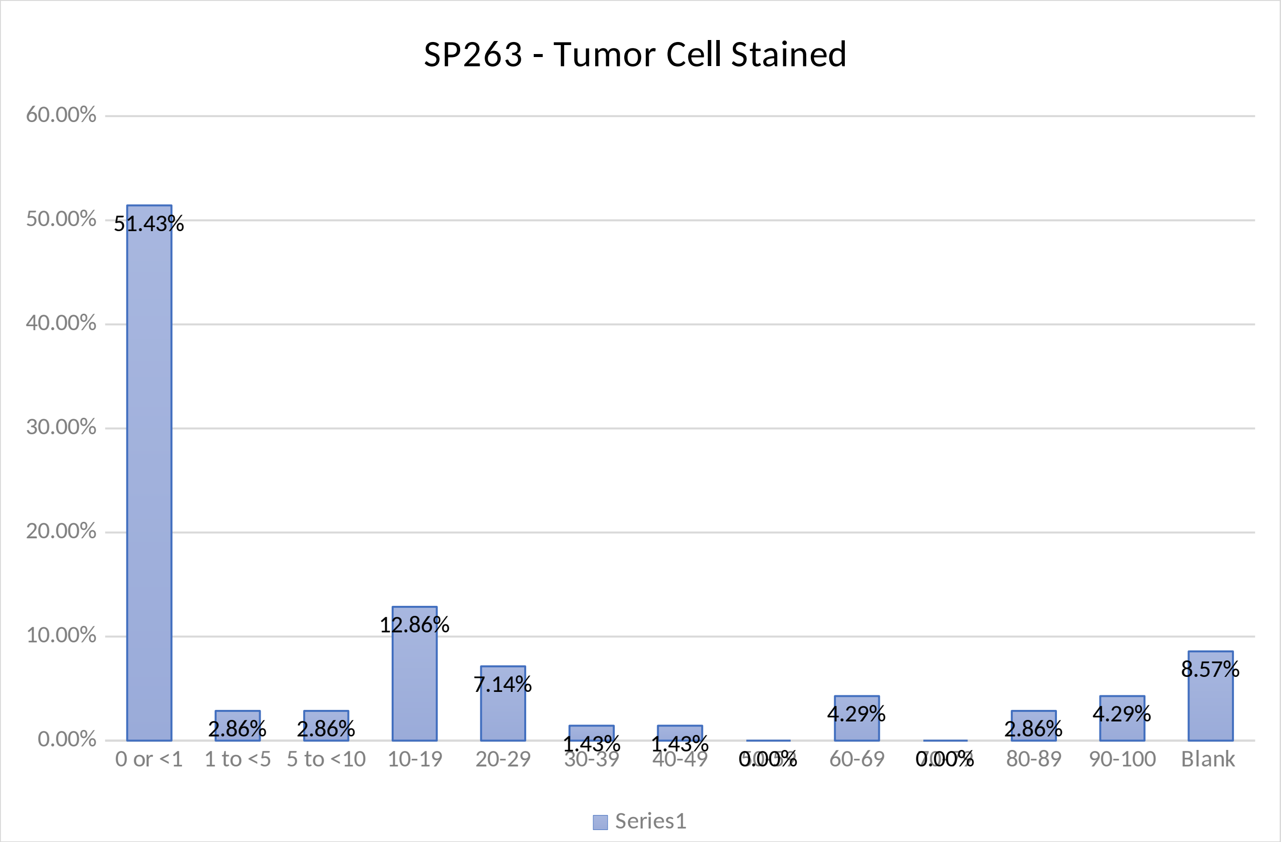
SP263 - Tumor Cell Stained
60.00%
50.00%
40.00%
30.00%
20.00%
10.00%
0.00%
51.43%
2.86%
2.86%
12.86%
7.14%
1.43%
1.43%
0.00%
4.29%
0.00%
2.86%
4.29%
8.57%
0 or <1
1 to <5
5 to <10
10-19
20-29
30-39
40-49
60-69
80-89
90-100
Blank
Series1
